# Friedreich's ataxia: the vicious circle hypothesis revisited

**DOI:** 10.1186/1741-7015-9-112

**Published:** 2011-10-11

**Authors:** Aurélien Bayot, Renata Santos, Jean-Michel Camadro, Pierre Rustin

**Affiliations:** 1 Inserm, U676, Physiopathology and Therapy of Mitochondrial Diseases Laboratory, CHU - Hôpital Robert Debré, 48, boulevard Sérurier, F-75019 Paris, France; 2 Faculté de médecine Denis Diderot, Université Paris-Diderot, IFR02, 16, rue Henri Huchard, F-75018, Paris, France; 3 Institut Jacques Monod (UMR 7592 CNRS-Université Paris-Diderot), Mitochondria, Metals and Oxidative Stress Laboratory, Bâtiment Buffon - 15, rue Hélène Brion, F-75205 Paris, Cedex 13, France

## Abstract

Friedreich's ataxia, the most frequent progressive autosomal recessive disorder involving the central and peripheral nervous systems, is mostly associated with unstable expansion of GAA trinucleotide repeats in the first intron of the *FXN *gene, which encodes the mitochondrial frataxin protein. Since *FXN *was shown to be involved in Friedreich's ataxia in the late 1990s, the consequence of frataxin loss of function has generated vigorous debate. Very early on we suggested a unifying hypothesis according to which frataxin deficiency leads to a vicious circle of faulty iron handling, impaired iron-sulphur cluster synthesis and increased oxygen radical production. However, data from cell and animal models now indicate that iron accumulation is an inconsistent and late event and that frataxin deficiency does not always impair the activity of iron-sulphur cluster-containing proteins. In contrast, frataxin deficiency appears to be consistently associated with increased sensitivity to reactive oxygen species as opposed to increased oxygen radical production. By compiling the findings of fundamental research and clinical observations we defend here the opinion that the very first consequence of frataxin depletion is indeed an abnormal oxidative status which initiates the pathogenic mechanism underlying Friedreich's ataxia.

## Background

**Figure 1:**
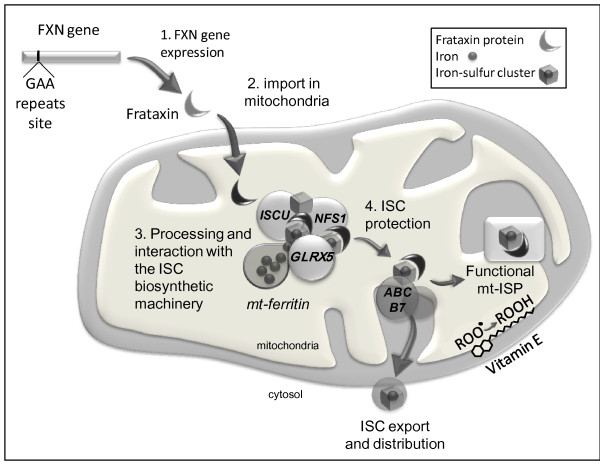
**Frataxin function in the mitochondria**. The schema illustrates the iron-sulphur cluster (ISC) biosynthesis machinery present in the mitochondrial matrix encompassing the ISCU-NFS1 protein complex associating glutaredoxin 5 (GLRX5) with the frataxin protein. It makes use of iron possibly delivered by the mitochondrial ferritin to synthesize ISC also distributed among several of the mitochondrial proteins (including several membrane-bound respiratory chain components, complexes I, II and III and the matrix-soluble aconitase). In addition to its role in the biogenesis of ISC, the frataxin protein might be associated with ISC after their synthesis. The detoxifying role of vitamin E in the mitochondrial inner membrane is also indicated. ISP, ISC-containing protein; mt, mitochondrial.

Friedreich's ataxia (FA), the most prevalent form of autosomal recessive cerebellar ataxia in Caucasians, is characterised by progressive ataxia and dysarthria [1]. The symptoms usually become apparent around puberty, although onset may occur much later in life (> 60 years old). The neurological features include sensory neuropathy, deep sensory impairment, signs of pyramidal tract involvement and progressive cerebellar dysfunction. The nonneurological manifestations vary, but among them hypertrophic cardiomyopathy is common. Diabetes mellitus occurs in approximately one-third of FA patients [1]. FA therefore appears to be a rather heterogeneous disorder. In the vast majority of cases, it is caused by a GAA trinucleotide repeat expansion in the first intron of the frataxin-encoding gene (*FXN*), which results in decreased gene expression and partial loss of function of the frataxin protein in the mitochondrial matrix [2]. Frataxin has been shown to interact with the iron-sulphur cluster (ISC) assembly machinery [3] (Figure 1). Frataxin loss of function therefore can result in ISC-containing protein (ISP) deficiency, decreasing aconitase and mitochondrial respiratory chain activity [4], but it also results in hypersensitivity to oxidative stress [5,6] and accumulation of iron in affected organs [7].

Except perhaps for gene-targeting therapies, to develop rational treatments for FA, we need to better elucidate the actual mechanism underlying the disease pathophysiology. On the basis of recent studies of various conditions in many different organisms (from microorganisms to humans), including human diseases originating from mutations in genes functionally related to *FXN*, we tried to reconcile the various pathogenic manifestations resulting from frataxin depletion and argue for a prominent and early role of impaired responses to oxidative insults in FA.

### The vicious circle hypothesis

**Figure 2:**
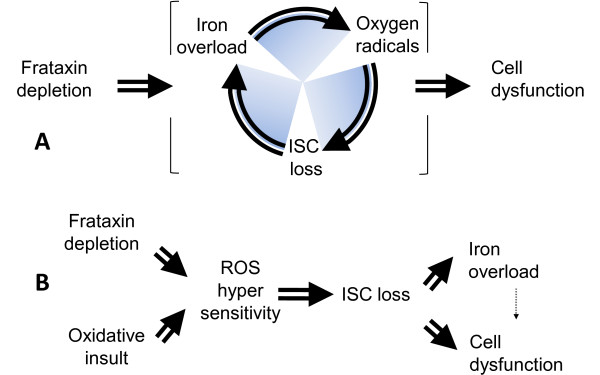
**The vicious circle hypothesis revisited in Friedreich's ataxia**. (A) According to the vicious circle hypothesis, frataxin depletion results in impaired iron-sulphur cluster synthesis and/or stability with intramitochondrial accumulation of reactive iron. Reactive iron promotes Fenton chemistry, producing superoxide and hydrogen peroxide, which in turn destroys more iron-sulphur clusters. ISC, iron-sulphur cluster. (B) In frataxin-depleted cells, deficient signalling of antioxidant defences sensitises the frataxin-free iron-sulphur clusters to reactive oxygen species. This antioxidant sensitisation process results in intramitochondrial iron accumulation, mostly as amorphous nonreactive precipitates. ROS, reactive oxygen species.

The cellular consequences of frataxin loss of function were initially described as faulty iron handling, impaired ISC synthesis and increased reactive oxygen species production [4]. We and others hypothesized that a vicious circle might link these three abnormalities (Figure 2A) and that targeting any of the three would consequently be as effective in slowing disease progression as targeting one or both of the other two abnormalities.

### Frataxin deficiency is not consistently associated with impaired iron-sulphur cluster synthesis or stability

In 1997, we reported the deficiency of ISP activity as the signature of frataxin depletion in humans [4]. Most tissues investigated, including skeletal muscle, lymphocytes and skin fibroblasts, were spared, however, and the deficiency was observed only in the heart and reported later in postmortem brain tissue. Since that initial observation, a series of remarkable works by Lill's group [3] carried out on frataxin-lacking yeast have conclusively demonstrated that frataxin has the capacity to participate in ISC synthesis through its interaction with the biosynthesis machinery.

However, in a number of conditions, frataxin depletion was not found to be associated with decreased activity of ISPs. In particular, as noted above, studies of frataxin-depleted cultured human skin fibroblasts, circulating lymphocytes or lymphoblastoid cell lines from FA patients found no decrease in ISPs [4], although the frataxin content was < 20% of the control value. Nevertheless, these cells have a phenotype that responds abnormally to a whole range of oxidative insults [6,8,9]. Studies of *Saccharomyces cerevisiae *also identified conditions under which, even in the total absence of frataxin, a significant synthesis of ISCs took place [10]. Thus, ISCs in frataxin-lacking *S. cerevisiae *were still synthesized when the cultures were protected from oxygen exposure (> 55% for 4Fe-4S clusters as compared to control yeast) [10]. This established that the function of frataxin in ISC synthesis and/or stability is dispensable under these conditions, as it is in a number of organisms, including Archaea and Gram-positive bacteria [11], and might be related to protection from oxygen-derived components.

### Intramitochondrial iron accumulation is a late event

As early as 1997, the intramitochondrial accumulation of iron was reported in the frataxin-lacking yeast, which led to the suggestion that frataxin regulates the export of iron from the mitochondrial matrix [12]. This echoed the even earlier observation of iron accumulation in postmortem human brain tissue [7]. Because reduced iron readily triggers deleterious Fenton chemistry, we and others have proposed that iron could be instrumental in the pathophysiological process that results in FA [13]. However, except for some circumstantial reports [5], attempts to detect significant mitochondrial iron accumulation in frataxin-depleted human fibroblasts and lymphoblasts, as well as changes in mitochondrial labile (reactive) iron, have failed [14]. Mitochondrial iron accumulates in frataxin-depleted cells only as a consequence of severely diminished ISC synthesis, resulting in amorphous, nonreactive, intramitochondrial precipitates of nanoparticles of ferric phosphate [15]. Accordingly, in mouse tissues lacking frataxin, mitochondrial iron deposition is a late event that follows the loss of ISPs [16]. Together these data suggest that mitochondrial iron accumulation is not an instrumental factor in the early steps of FA pathogenesis.

### Frataxin depletion does not cause overproduction of superoxides

On the basis of the putative respiratory chain impairment and/or the iron-associated Fenton chemistry presumably resulting from iron mishandling, it has been inferred that frataxin depletion should result in overproduction of oxidant species. This inference was supported by the report of increased oxidative insult to DNA in FA patients [17]. However, under a number of conditions, no increase in oxidative species could be detected, and the mitochondria from frataxin-depleted cells (that is, fibroblasts) did not significantly produce more superoxides (assessed using the MitoSOX Red reagent; Invitrogen, Carlsbad, CA, USA than control cells, and targeting superoxides in frataxin-depleted human cells using Mn(III) tetrakis (4-benzoic acid) porphyrin chloride failed to restore a normal phenotype in these cells (P Rustin, unpublished data).

### Frataxin deficiency consistently results in hypersensitivity to oxygen radicals

Numerous studies using various models have established that partial or complete frataxin deficiency results in varying degrees of respiratory chain impairment. Superoxide production by the mitochondria is mostly proportional to the electron flow and to the reduction of critical components of the chain, whereas superoxide elimination essentially depends on superoxide dismutase activity. The very severe impairments in respiratory chain and Krebs cycle activity associated with the absence of frataxin presumably result in decreased superoxide production. Underestimation of the consequences of complete frataxin deficiency has led to the apparently paradoxical conclusion that oxidative stress may not play a major role in FA pathology [18]. In contrast, in affected human tissues and a number of FA models, respiratory chain activity is only partially affected and the superoxide production machinery is therefore largely intact. In human fibroblasts with low frataxin content, we found a slight but consistent (< 30%) elevation in basal superoxide dismutase activity [9], indicating that the oxidative status might be slightly abnormal, at least under conditions where the respiratory chain is fully active [19]. Similarly, in contrast to mice lacking frataxin [18], mice with partial loss of frataxin show increased signs of lipoperoxidation [20].

Studies of the antioxidant pools in frataxin-depleted cultured human cells have shown significant decreases in reduced glutathione along with actin stress fibre disorganization [21], but this reduction in antioxidant pools have little or no impact on the cells under basal conditions. However, these cells and their iron-sulphur proteins have exquisite sensitivity to a wide range of experimental oxidative insults [9], which often trigger rapid apoptosis [5,6,8]. This hypersensitivity was found in all frataxin-deficient cell types and organisms independently of abnormalities in mitochondrial ISP or iron content (Table 1). It was observed in connection with endogenous insults (respiratory chain blockade) and exogenous insults (various chemicals that generated oxidant species) as well. These findings suggest impairment of a set of antioxidant defences as opposed to one specific enzyme. Accordingly, impaired signalling of phase II antioxidant defences has been observed in frataxin-depleted human cells [9].

**Table 1:** Consequences of frataxin depletion in various organisms^a^

Organism	Increased ROS production/low glutathione	Increased peroxidation products	Hypersensitivity to oxidative insults	Loss of ISP activity	Mitochondrial iron overload	References
*Saccharomyces* *cerevisiae *yeast	+/++	+++	**+++**	+++	+++	[19]
*Arabidopsis* *thaliana *plant	+	n.d.	**+++**	+++	++	[69]
*Caenorhabditis* *elegans *worm	n.d.	n.d.	**+++**	+++	n.d.	[50]
*Drosophila melanogaster *fly	(+)	n.d.	**+++**	+++	n.d.	[70]
*Mus musculus *mouse (humanised)	n.d.	+++	n.d.	+++	-	[20]
*Homo sapiens* heart	n.d.	n.d.	n.d.	+++	++	[4]
*Homo sapiens *lymphoblasts	n.d.	-	**+++**	-	-	[14]
*Homo sapiens *skin fibroblasts	+	-	**+++**	-	-	[9]

^a^ROS: reactive oxygen species; ISP: iron-sulphur cluster-containing protein; +++: hallmark of the disease; ++: present in most cases; +: present only in a subset of patients or late in the course of the disease; (+): reported occasionally; -: usually absent; n.d., not determined.

This impairment is related to abnormalities in the nuclear factor erythroid 2-related factor 2 (Nrf2) transcription factor, which may result from disorganization of the actin stress filaments and abnormalities in the redox status of frataxin-deficient cells [9]. Concomitant impairments in related signalling pathways for mitochondrial components in response to the abnormal redox status of frataxin-depleted cells have also been reported [22].

### Lessons from clinical practice

Interestingly, myopathy without ataxia associated with impaired ISC synthesis was described recently [23]. This condition is due to mutation in the gene encoding the ISC scaffold protein (*ISCU*) in which differential splicing among tissues largely explains the tissue specificity [24]. Although recent data from Isaya's group [25] show that two frataxin isoforms do exist and have distinct functional properties regarding iron storage and/or ISC synthesis [25], expression of the protein appears widespread in the organism, and both isoforms are supposedly similarly affected by GAA expansion in the gene. Accordingly, frataxin loss of function appears to be widespread in FA patients. Yet, the consequences are mostly observed in the brain and the heart, and primary myopathy is not a consistent or frequent feature of this disease [26].

Because frataxin has been reported to interact directly with the NFS1-ISCU protein complex during ISC biosynthesis [3] (Figure 1), a certain level of overlap might be predicted, at least in tissues where the *NFS1 *gene mutation causes a phenotype. The absence of such overlap is thus not easy to reconcile with the fact that the two proteins are involved in the same step of ISC biosynthesis *in vivo *(Table 2). A similar observation stands true regarding glutaredoxin 5 (an assembly factor for cellular ISC) [27] (Figure 1), as a mutation in the exon 1 of the *GLRX5 *gene, putatively leading to a deleterious splicing defect, results in sideroblastic anaemia without ataxia and no overlap with the FA phenotype [28].

**Table 2:** Cardinal clinical symptoms associated with deficiencies in frataxin, vitamin E, ABCB7 (mitochondrial iron overload), GLRX5 (impaired ISC synthesis) and ISCU (impaired ISC synthesis)^a^

Clinical symptoms	Frataxin deficiency	Vitamin E deficiency	ABCB7 deficiency	GLRX5 deficiency	ISCU deficiency
Ataxia	**+++**	**+++**	**+++**	**-**	**-**
Cardiomyopathy	**+**	**+**	**-**	**-**	**-**
Diabetes mellitus	+	(+)	-	**+**	**-**
Myopathy	(+)	(+)	-	**-**	**+++**
Sideroblastic anaemia	-	-	+++	**+++**	**-**
Hepatosplenomegaly	-	-	-	**++**	**-**
Lactic acidosis	(+)	(+)	-	**-**	**+++**

^a^+++: hallmark of the disease; ++: present in most cases; +: present only in a subset of patients or late in the course of the disease; (+): reported occasionally; -: usually absent. Disease symptoms shared with Friedreich's ataxia are represented using a similar grey colour.

Accumulation of mitochondrial iron and decreased cytosolic iron associated with the *ABCB7 *mutations, mostly missense mutations changing amino acids in the C-terminal end of the transmembrane domain of the protein, results in cerebellar ataxia, albeit different from FA, and includes sideroblastic anaemia [29,30]. This ABC transporter is involved in ISC export from the mitochondria to the cytosol [31] (Figure 1). Yeast studies showed preserved mitochondrial ISP activity despite intramitochondrial iron accumulation [32]. However, as with frataxin depletion, hypersensitivity to oxidative stress has been reported in *ABCB7 *mutants [33], suggesting that a common mechanism resulting in impaired handling of reactive oxygen species may be involved at some point in both types of ataxia.

Finally, the striking similarity of the symptoms in vitamin E deficiency and frataxin deficiency [34], both encompassing cerebellar ataxia with inconsistent cardiomyopathy [35], suggests similar cellular consequences of frataxin and vitamin E depletion. Vitamin E acts chiefly as a membrane antioxidant, in concert with ubiquinone, and plays no role in ISC biosynthesis [36] (Figure 1).

Thus, a comparison of the various clinical phenotypes (Table 2) is consistent with, though obviously not proving *per se*, the view [37] emerging from studies of various situations and models (Table 1) that frataxin depletion results primarily in increased sensitivity to oxidative stress (Figure 2B).

## Discussion

Since the discovery of the gene mutation responsible for FA, major advances have been made in our understanding of this disease, despite the difficulties encountered in gaining access to affected tissue in humans, especially the brain. In particular, FA can now be regarded as a true mitochondrial disease similar to a number of mitochondrial ataxias due to different genetic mechanisms [38]. The respective roles of deficient respiratory chain function (decreased ATP synthesis) and impaired oxygen handling in the clinical course remain to be determined in most of these diseases. However, in the case of FA, impaired oxygen handling appears to be crucial in the cascade of events determining the onset of the symptoms and fits the progressive degenerative course of the disease. Obviously, emphasizing the role of hypersensitivity to oxygen in triggering FA does not shed any light on the undisputed contributory role of frataxin in ISC synthesis and/or stability [39,40]. Yet, available data indicate that both processes can be dissociated under a number of conditions in different models, including humans. Unfortunately, the molecular mechanism linking frataxin function to this hypersensitivity to oxygen is not yet established, similarly to the exact role of the protein in ISC synthesis, which is still a matter of intense debate. Frataxin has been claimed to activate import of iron into the cell [41] and to chaperone iron [42,43] or ISC [44,45] to act as a partner for [46] or inhibitor of [47] ISC synthesis. How frataxin shortage in mitochondria results in impaired signalling of antioxidant defences in the cell cytosol has yet to be elucidated. So far, it is only known that frataxin deficiency in human cells interferes with the redox status of the cell [21], thus presumably impairing the function of the Nrf2 [9] and possibly PGC1α [48] transcription factors.

Hypersensitivity to oxidant insult is a consistent feature of human cells [5,6,8,9] and animal models [49,50] with low frataxin content (Table 1). This observation should not be confused with an increased production of oxidative species and peroxidised products, yet has frequently been observed and reported (in about 100 papers), which either may not be observed, especially if respiratory chain activity is too severely depressed, or may not be accumulated if produced at low levels. Finally, in FA patients, oxidative markers and/or antioxidant enzymes are also modified in response to frataxin depletion [17,51-54], which was found to be an incentive to trial antioxidant molecules in this disease [55-57].

Several ongoing clinical trials are evaluating interventions that target various steps in the pathogenic process: impaired frataxin synthesis, oxidative insults and iron accumulation [58]. Idebenone, a short-chain coenzyme Q10 homologue, has initially been reported to be effective (at a dosage of 5 to 20 mg/kg/day) in preventing cardiac hypertrophy in most patients [59], while having (at this dosage) little or no effect on the neurological abnormalities [19]. Interestingly, while studying endomyocardial biopsies from a young patient with FA before and after idebenone treatment, we found that the drug largely restored the activity of ISPs [60]. Since idebenone is a potent antioxidant, this finding suggests that loss of ISP activity *in vivo *is due chiefly to increased oxidative degradation, and not to impaired synthesis, of these proteins. In this context, we may wonder why antioxidant therapies (for example, idebenone, coenzyme Q10) have such a limited impact on the neurological disease expression and/or course in FA. Indeed, recent widely based studies carried out in Europe (the MICONOS (Mitochondrial Protection with Idebenone in Cardiac Or Neurological Outcome Study) trial, comprising 232 patients with 162 on idebenone, mostly adults, at 13 centres; Andrews WT unpublished communication from the FARA meeting in Strasbourg, France, June 2011) and in the United States (the IONIA (Idebenone Effects on Neurological International Cooperative Ataxia Rating Scale Assessments) trial; 70 patients) [61] did not confirm the previous reports of the beneficial effects of idebenone in this disease. However, as mentioned by the initiators of the MICONOS trial themselves, the results of the available studies, even if not statistically significant, still indicate a trend toward a positive effect of idebenone (as compared to placebo). A possibly underestimated factor that might prevent the results of such studies from reaching statistical significance in their relatively small cohorts is the occurrence of responsive and unresponsive patients reported in early FA patients treated with idebenone [59], as has been true for a number of other mitochondrial diseases [62]. Conversely, the negative conclusion of these latter trials, mainly carried out with adults, might be related to the age of the patients. Accordingly, even more recently this year, an open-label extension of the IONIA study (IONIA-E trial; 68 patients) concluded that idebenone may offer a therapeutic benefit to paediatric FA patients by stabilizing overall neurological function and improving fine motor skills and speech [63]. There is no definite answer to this question; however, a number of indications suggest that intervention might be much too late. Indeed, it is a frequent observation in neurological diseases caused by gene mutations (nuclear or mitochondrial) encoding mitochondrial proteins that symptoms are subsequent to extended auto-amplifying cell death resulting from mitochondrial dysfunction rather than from mitochondrial dysfunction itself (for example, ATP decrease, metabolic blockade). This can be observed in a number of animal models where early, partial or tissue-specific inactivation of such genes (*Tfam *and *Aif*) results in a delayed neurological phenotype despite early mitochondrial dysfunction [64,65]. Thus, Tfam-depleted neurons, despite severe respiratory chain deficiency, are viable for one month in the mouse without showing signs of the neurodegeneration which precedes neurological symptoms [64]. Similarly, despite early detectable complex I deficiency in the brain, the *Harlequin *mouse with depleted Aif protein only manifests significant symptoms after several weeks or months of life in most individuals [65]. Likewise, frataxin gene loss of function in FA, although it occurs early in embryogenesis, has a neurological impact several years later. Thus, the onset of neurological symptoms associated with impaired mitochondrial function might follow the loss of neurons rather than mitochondrial dysfunction *per se*. Accordingly, any therapy aimed at counteracting mitochondrial dysfunction, regardless of the strategy used (modulating gene expression, gene therapy or pharmacological therapy) would be best tested if it preceded disease initiation.

## Conclusions

The present compilation of recent data on the pathological cascade in FA argues in favour of continuing experimentation with antioxidants in FA, despite the deceptive results of the most recent idebenone clinical trials, but care should be taken to focus on selected cohorts of patients within the presumed therapeutic window. Additional therapeutic strategies aimed at counteracting hypersensitivity to reactive oxygen species should also be developed. In keeping with this, pioglitazone, a peroxisome proliferator-activated receptor γ ligand that improves natural antioxidant defences (including frataxin), is presently being under trial in our hospital in France (two-year phase III trial ending in 2013). Other approaches aimed at increasing frataxin levels, histone deacetylase inhibitors [66] and nonerythropoietic derivatives of erythropoietin [67] may also result in decreased cellular hypersensitivity to reactive oxygen species, as a direct correlation has been shown between this hypersensitivity and the actual level of frataxin, from depletion to overexpression [68]. We hope that some of these promising compounds will prove effective in halting the progression of FA.

## Competing interests

The authors declare that they have no competing interests.

## Authors' contributions

All authors participated in the conception of this opinion paper, which was written by PR. All authors read and approved the final manuscript.

## Pre-publication history

The pre-publication history for this paper can be accessed here:

http://www.biomedcentral.com/1741-7015/9/112/prepub
